# Supplementary material for: Hypothyroidism following Radiotherapy for Head and Neck Cancer: A Systematic Review of the Literature and Opportunities to Improve the Therapeutic Ratio
Source: Cancers (Basel). 2023 Aug 29;15(17):4321. doi: 10.3390/cancers15174321 (PMC10486996; doi:10.3390/cancers15174321)
Supplement: Supplementary file 1 [file cancers-15-04321-s001.zip › cancers-2537661-supplementary.docx]

**Supplemental Table S1.** List of all included articles

| **PMID** | **First Author** | **Year** | **Reported Median or Mean Population Size** | **Journal** | **Title** |
| --- | --- | --- | --- | --- | --- |
| 17998645 | Aich RK | 2005 | 187 | J Cancer Res Ther | Iatrogenc hypothyroidism: a consequence of external beam radiotherapy to the head & neck malignancies. |
| 24885512 | Akgun Z | 2014 | 100 | Radiat Oncol | V30 as a predictor for radiation-induced hypothyroidism: a dosimetric analysis in patients who received radiotherapy to the neck. |
| 26975210 | Alba JR | 2016 | 241 | J Laryngol Otol | Hypothyroidism in patients treated with radiotherapy for head and neck carcinoma: standardised long-term follow-up study. |
| 19041504 | Alkan S | 2008 | 75 | Otolaryngol Head Neck Surg | Thyroid dysfunction after combined therapy for laryngeal cancer: a prospective study |
| 17084554 | Alterio D | 2007 | 73 | Int J Radiat Oncol Biol Phys | Thyroid disorders in patients treated with radiotherapy for head-and-neck cancer: a retrospective analysis of seventy-three patients. |
| 8957119 | August M | 1996 | 35 | J Oral Maxillofac Surg | Complications associated with therapeutic neck radiation |
| 22000746 | Bakhshandeh M | 2012 | 65 | Int J Radiat Oncol Biol Phys | Evaluation of thyroid disorders during head-and-neck radiotherapy by using functional analysis and ultrasonography. |
| 22583606 | Bakhshandeh M | 2013 | 50 | Int J Radiat Oncol Biol Phys | Normal tissue complication probability modeling of radiation-induced hypothyroidism after head-and-neck radiation therapy. |
| 21768665 | Banipal R | 2011 | 53 | Indian J Cancer | Thyroid diseases as a sequelae following treatment of head and neck cancer. |
| 24210455 | Bernát L | 2014 | 83 | J Craniomaxillofac Surg | Hypothyroidism after radiotherapy of head and neck cancer. |
| 17446000 | Bhandare N | 2007 | 312 | Int J Radiat Oncol Biol Phys | Primary and central hypothyroidism after radiotherapy for head-and-neck tumors. |
| 22717243 | Boomsma MJ | 2012 | 105 | Int J Radiat Oncol Biol Phys | A prospective cohort study on radiation-induced hypothyroidism: development of an NTCP model. |
| 1951886 | Buisset E | 1991 | 32 | Am J Surg | Hypothyroidism following combined treatment for hypopharyngeal and laryngeal carcinoma. |
| 7968176 | Cannon CR | 1994 | 62 | Laryngoscope | Hypothyroidism in head and neck cancer patients: experimental and clinical observations. |
| 18468330 | Cetinayak O | 2008 | 378 | Tumori | Assessment of treatment-related thyroid dysfunction in patients with head and neck cancer. |
| 36336109 | Chow JCH | 2022 | 488 | Radiother Oncol | Post-radiation primary hypothyroidism in patients with head and neck cancer: External validation of thyroid gland dose-volume constraints with long-term endocrine outcomes. |
| 25476839 | Chyan A | 2014 | 123 | Radiat Oncol | Dosimetric predictors of hypothyroidism in oropharyngeal cancer patients treated with intensity-modulated radiation therapy |
| 11597798 | Colevas AD | 2001 | 118 | Int J Radiat Oncol Biol Phys | Hypothyroidism incidence after multimodality treatment for stage III and IV squamous cell carcinomas of the head and neck. |
| 19577867 | Diaz R | 2010 | 168 | Int J Radiat Oncol Biol Phys | Hypothyroidism as a consequence of intensity-modulated radiotherapy with concurrent taxane-based chemotherapy for locally advanced head-and-neck cancer. |
| 7554340 | Donnelly MJ | 1995 | 27 | Clin Otolaryngol Allied Sci | Thyroid dysfunction following combined therapy for laryngeal carcinoma. |
| 29428369 | El-Shebiney M | 2018 | 78 | J Egypt Natl Canc Inst | Radiotherapeutic factors affecting the incidence of developing hypothyroidism after radiotherapy for head and neck squamous cell cancer. |
| 28532605 | Fan CY | 2017 | 14893 nasopharynx and 16105 other HN cancer pts, respectively | Radiother Oncol | Risk of hypothyroidism among patients with nasopharyngeal carcinoma treated with radiation therapy: A Population-Based Cohort Study. |
| 25818629 | Fujiwara M | 2015 | 116 | J Radiat Res | The threshold of hypothyroidism after radiation therapy for head and neck cancer: a retrospective analysis of 116 cases. |
| 15923797 | Garcia-Serra A | 2005 | 206 | Am J Clin Oncol | Thyroid function should be monitored following radiotherapy to the low neck. |
| 1410587 | Grande C | 1992 | 221 | Radiother Oncol | Hypothyroidism following radiotherapy for head and neck cancer: multivariate analysis of risk factors. |
| 27511845 | Harris J | 2016 | 10 | Int J Radiat Oncol Biol Phys | Free Thyroid Transfer: A Novel Procedure to Prevent Radiation-induced Hypothyroidism. |
| 27862538 | Harris J | 2017 | 10 | Head Neck | Free thyroid transfer: Short-term results of a novel procedure to prevent post-radiation hypothyroidism. |
| 31560840 | Huang C | 2019 | 345 | Cancer Med | Thyroid dose-volume thresholds for the risk of radiation-related hypothyroidism in nasopharyngeal carcinoma treated with intensity-modulated radiotherapy-A single-institution study |
| 23999842 | Huang S | 2013 | 97 | Med Oncol | Hypothalamic-pituitary-thyroid dysfunction induced by intensity-modulated radiotherapy (IMRT) for adult patients with nasopharyngeal carcinoma |
| 34738883 | Inoue E | 2021 | 52 | Acta Otolaryngol | Factors predictive of the development of hypothyroidism after intensity-modulated radiation therapy for pharyngeal cancer. |
| 29277803 | Ishibashi N | 2018 | 24 | Anticancer Res | Computed Tomography Density Change in the Thyroid Gland Before and After Radiation Therapy. |
| 36186696 | Jia-Mahasap B | 2022 | 62 | Rep Pract Oncol Radiother | Incidence of radiation-induced hypothyroidism following head and neck irradiation: a single-center analysis. |
| 32051897 | Kamal M | 2019 | 360 | Adv Radiat Oncol | Radiation-Induced Hypothyroidism After Radical Intensity Modulated Radiation Therapy for Oropharyngeal Carcinoma. |
| 30594401 | Katna R | 2019 | 6 | Am J Otolaryngol | Free thyroid transfer to anterolateral thigh for prevention of radiation induced hypothyroidism: An initial experience. |
| 24482412 | Kim MY | 2014 | 114 | Jpn J Clin Oncol | Dose-volumetric parameters for predicting hypothyroidism after radiotherapy for head and neck cancer. |
| 32016495 | Kinclová I | 2020 | 83 | Strahlenther Onkol | Model-based calculation of thyroid gland normal tissue complication probability in head and neck cancer patients after radiation therapy. |
| 12798403 | Koc M | 2003 | 47 | Oral Oncol | A prospective evaluation of early thyroid dysfunction on completion of neck radiotherapy. |
| 19307948 | Koc M | 2009 | 63 | Am J Clin Oncol | Thyroid dysfunction in patients treated with radiotherapy for neck. |
| 21328003 | Kumar S | 2011 | 33 | Eur Arch Otorhinolaryngol | The incidence of thyroid dysfunction following radiotherapy for early stage carcinoma of the larynx |
| 28448252 | Kumari S | 2017 | 383 | Rambam Maimonides Med J | Hypothyroidism in Carcinoma of the Tongue with Adjuvant Treatment |
| 11033194 | Kumpulainen EJ | 2000 | 72 | Radiother Oncol | Hypothyroidism after radiotherapy for laryngeal cancer |
| 8627999 | Kuten A | 1996 | 43 | J Surg Oncol | Postradiotherapy hypothyroidism: radiation dose response and chemotherapeutic radiosensitization at less than 40 Gy. |
| 33609046 | Leddon JL | 2021 | 139 | Laryngoscope | Hypothyroidism in Head and Neck Squamous Cell Carcinoma Patients Receiving Radiotherapy With or Without Immune Checkpoint Inhibitors. |
| 18723296 | Lee A | 2009 | 422 | Int J Radiat Oncol Biol Phys | Major late toxicities after conformal radiotherapy for nasopharyngeal carcinoma-patient- and treatment-related risk factors |
| 27235379 | Lee V | 2016 | 149 | Clin Oncol (R Coll Radiol) | Dosimetric Predictors of Hypothyroidism After Radical Intensity-modulated Radiation Therapy for Non-metastatic Nasopharyngeal Carcinoma |
| 29302704 | Lertbutsayanukul C | 2018 | 209 | Strahlenther Onkol | A randomized phase III study between sequential versus simultaneous integrated boost intensity-modulated radiation therapy in nasopharyngeal carcinoma |
| 29750261 | Lertbutsayanukul C | 2018 | 178 | J Radiat Res | Validation of previously reported predictors for radiation-induced hypothyroidism in nasopharyngeal cancer patients treated with intensity-modulated radiation therapy, a post hocanalysis from a Phase III randomized trial |
| 29580932 | Lin AJ | 2019 | 77 | Med Dosim | Postradiation hypothyroidism in head and neck cancers: A Department of Veterans Affairs single-institution case-control dosimetry study. |
| 21054241 | Lin Z | 2011 | 45 | Thyroid | A longitudinal study on the radiation-induced thyroid gland changes after external beam radiotherapy of nasopharyngeal carcinoma. |
| 23559564 | Lin Z | 2014 | 65 | Head Neck | Longitudinal study on the correlations of thyroid antibody and thyroid hormone levels after radiotherapy in patients with nasopharyngeal carcinoma with radiation-induced hypothyroidism. |
| 24283561 | Lin Z | 2013 | 50 | J Med Imaging Radiat Oncol | Evaluation of clinical hypothyroidism risk due to irradiation of thyroid and pituitary glands in radiotherapy of nasopharyngeal cancer patients. |
| 29985952 | Lin Z | 2018 | 56 | PLoS One | Pattern of radiation-induced thyroid gland changes in nasopharyngeal carcinoma patients in 48 months after radiotherapy |
| 27905164 | Ling S | 2017 | 102 | Head Neck | Correlative study of dose to thyroid and incidence of subsequent dysfunction after head and neck radiation. |
| 17364370 | Lo Galbo AM | 2007 | 156 | Acta Otolaryngol | The prevalence of hypothyroidism after treatment for laryngeal and hypopharyngeal carcinomas: are autoantibodies of influence? |
| 18766358 | Lo Galbo AM | 2009 | 32 | Eur Arch Otorhinolaryngol | Detecting hypothyroidism after treatment for laryngeal or hypopharyngeal carcinomas: a nationwide survey in The Netherlands. |
| 36217182 | Lu HH | 2022 | 52 | Cancer Imaging | Early post-treatment (18)F-FDG PET/CT for predicting radiation-induced hypothyroidism in head and neck cancer. |
| 27885853 | Luo R | 2016 | 164 | Br J Radiol | Nomogram for radiation-induced hypothyroidism prediction in nasopharyngeal carcinoma after treatment |
| 29776390 | Luo R | 2018 | 174 | BMC Cancer | Development of a normal tissue complication probability (NTCP) model for radiation-induced hypothyroidism in nasopharyngeal carcinoma patients |
| 30191868 | McDowell | 2018 | 107 | Int J Radiat Oncol Biol Phys | Long-Term Late Toxicity, Quality of Life, and Emotional Distress in Patients With Nasopharyngeal Carcinoma Treated With Intensity Modulated Radiation Therapy |
| 11753963 | Mercado G | 2001 | 143 | Cancer | Hypothyroidism: a frequent event after radiotherapy and after radiotherapy with chemotherapy for patients with head and neck carcinoma. |
| 26362315 | Mulholland GB | 2015 | 95 | J Otolaryngol Head Neck Surg | Optimal detection of hypothyroidism in early stage laryngeal cancer treated with radiotherapy. |
| 23996654 | Murthy V | 2014 | 122 | Head Neck | Hypothyroidism after 3-dimensional conformal radiotherapy and intensity-modulated radiotherapy for head and neck cancers: prospective data from 2 randomized controlled trials. |
| 17043248 | Nelson M | 2006 | 155 | Arch Otolaryngol Head Neck Surg | Association between development of hypothyroidism and improved survival in patients with head and neck cancer. |
| 17023776 | Norris AA | 2006 | 169 | Am J Clin Oncol | Hypothyroidism when the thyroid is included only in the low neck field during head and neck radiotherapy. |
| 32971838 | Nowicka Z | 2020 | 108 | Cancers (Basel) | Radiation-Induced Hypothyroidism in Patients with Oropharyngeal Cancer Treated with IMRT: Independent and External Validation of Five Normal Tissue Complication Probability Models. |
| 12742272 | O'Meara | 2003 | 62 | Radiother Oncol | Follow-up of head and neck cancer patients post-radiotherapy |
| 17162132 | Ozawa H | 2007 | 35 | Am J Otolaryngol | Hypothyroidism after radiotherapy for patients with head and neck cancer. |
| 36408306 | Pal SK | 2022 | 95 | Cureus | Determining the Occurrence of Hypothyroidism Following Treatment With Radiation Therapy in Head and Neck Carcinoma Patients and the Associated Role of Risk Factors and Dose-Volume Histograms: A Prospective Study. |
| 22995588 | Peng L | 2012 | 545 | Radiother Oncol | A prospective, randomized study comparing outcomes and toxicities of intensity-modulated radiotherapy vs. conventional two-dimensional radiotherapy for the treatment of nasopharyngeal carcinoma |
| 33102218 | Peng L | 2020 | 616 | Front Oncol | A New Model for Predicting Hypothyroidism After Intensity-Modulated Radiotherapy for Nasopharyngeal Carcinoma. |
| 29709128 | Pil J | 2016 | 72 | B-ENT | The incidence of hypothyroidism after radiotherapy for head and neck cancer. |
| 31833166 | Plaat RE | 2020 | 128 | Head Neck | Onset of hypothyroidism after total laryngectomy: Effects of thyroid gland surgery and preoperative and postoperative radiotherapy. |
| 2414254 | Posner MR | 1985 | 100 | Int J Radiat Oncol Biol Phys | Treatment complications after sequential combination chemotherapy and radiotherapy with or without surgery in previously untreated squamous cell carcinoma of the head and neck. |
| 6708688 | Posner MR | 1984 | 43 | Laryngoscope | Incidence of hypothyroidism following multimodality treatment for advanced squamous cell cancer of the head and neck. |
| 31747379 | Prpic M | 2019 | 70 | Radiol Oncol | Dose-volume derived nomogram as a reliable predictor of radiotherapy-induced hypothyroidism in head and neck cancer patients. |
| 34528559 | Randhawa AS | 2021 | 45 | J Cancer Res Ther | Functional and biochemical changes in the thyroid gland following exposure to therapeutic doses of external beam radiotherapy in the head-and-neck cancer patients. |
| 35155788 | Ranta P | 2021 | 233 | Laryngoscope Investig Otolaryngol | Dysphagia, hypothyroidism, and osteoradionecrosis after radiation therapy for head and neck cancer. |
| 31742001 | Rao D | 2019 | 100 | Indian J Otolaryngol Head Neck Surg | Thyroid Dysfunction Following Management of Non-thyroid Head and Neck Cancers. |
| 34425512 | Ren W | 2021 | 145 | Phys Med | Dosiomics-based prediction of radiation-induced hypothyroidism in nasopharyngeal carcinoma patients |
| 23891099 | Rønjom MF | 2013 | 46 | Radiother Oncol | Hypothyroidism after primary radiotherapy for head and neck squamous cell carcinoma: normal tissue complication probability modeling with latent time correction. |
| 25629441 | Rønjom MF | 2015 | 198 | Acta Oncol | Variation of normal tissue complication probability (NTCP) estimates of radiation-induced hypothyroidism in relation to changes in delineation of the thyroid gland. |
| 26248025 | Rønjom MF | 2015 | 203 | Acta Oncol | External validation of a normal tissue complication probability model for radiation-induced hypothyroidism in an independent cohort. |
| 25503434 | Sachdev S | 2017 | 75 | Am J Clin Oncol | Thyroid V50 Highly Predictive of Hypothyroidism in Head-and-Neck Cancer Patients Treated With Intensity-modulated Radiotherapy (IMRT). |
| 7153098 | Samaan | 1982 | 110 | Int J Radiat Oncol Biol Phys | Hypothalamic, pituitary and thyroid dysfunction after radiotherapy to the head and neck |
| 1130929 | Shafer RB | 1975 | 61 | Arch Intern Med | Thyroid function after radiation and surgery for head and neck cancer. |
| 10807335 | Sinard RJ | 2000 | 136 | Arch Otolaryngol Head Neck Surg | Hypothyroidism after treatment for nonthyroid head and neck cancer. |
| 19360741 | Smith GL | 2009 | 5916 | Head Neck | Hypothyroidism in older patients with head and neck cancer after treatment with radiation: a population-based study. |
| 10884190 | Smolarz K | 2000 | 120 | Thyroid | Hypothyroidism after therapy for larynx and pharynx carcinoma. |
| 34771747 | Smyczynska U | 2021 | 98 | Cancers (Basel) | Prediction of Radiation-Induced Hypothyroidism Using Radiomic Data Analysis Does Not Show Superiority over Standard Normal Tissue Complication Models. |
| 28581397 | Sommat K | 2017 | 102 | Int J Radiat Oncol Biol Phys | Thyroid V40 Predicts Primary Hypothyroidism After Intensity Modulated Radiation Therapy for Nasopharyngeal Carcinoma |
| 22557780 | Srikantia N | 2011 | 45 | Indian J Med Paediatr Oncol | How common is hypothyroidism after external radiotherapy to neck in head and neck cancer patients? |
| 1443391 | Tami TA | 1992 | 100 | Am J Otolaryngol | Thyroid dysfunction after radiation therapy in head and neck cancer patients. |
| 29147388 | Tariq K | 2014 | 1116 | World J Oncol | Relationship of Gender and Smoking History in the Development of Hypothyroidism From Exposure to External Beam Radiation for Head and Neck Cancers. |
| 9308932 | Tell R | 1997 | 391 | Int J Radiat Oncol Biol Phys | Hypothyroidism after external radiotherapy for head and neck cancer |
| 15380571 | Tell R | 2004 | 264 | Int J Radiat Oncol Biol Phys | Long-term incidence of hypothyroidism after radiotherapy in patients with head-and-neck cancer. |
| 10225153 | Thorp MA | 1999 | 21 | Clin Otolaryngol Allied Sci | Parathyroid and thyroid function five years after treatment of laryngeal and hypopharyngeal carcinoma |
| 7836081 | Turner SL | 1995 | 84 | Int J Radiat Oncol Biol Phys | Thyroid dysfunction following radiotherapy for head and neck cancer. |
| 33775035 | Uddin N | 2021 | 100 | J Coll Physicians Surg Pak | Relation of External Beam Radiotherapy Dose with Subclinical Hypothyroidism in Patients undergoing Adjuvant Neck Radiation after Surgery for Squamous Cell Carcinoma of Head and Neck. |
| 17673817 | Ulger S | 2007 | 85 | Med Oncol | Incidence of hypothyroidism after radiotherapy for nasopharyngeal carcinoma. |
| 1938359 | Weissler MC | 1991 | 68 | Head Neck | Thyroid-stimulating hormone levels after radiotherapy and combined therapy for head and neck cancer. |
| 35170140 | Weng JJ | 2022 | 284 | Head Neck | Prognostic value of hypothyroidism in patients undergoing intensity-modulated radiation therapy for nasopharyngeal carcinoma. |
| 19596170 | Wu YH | 2010 | 408 | Int J Radiat Oncol Biol Phys | Hypothyroidism After Radiotherapy for Nasopharyngeal Cancer Patients |
| 30405757 | Xu Y | 2018 | 52 | Oncol Lett | A dosimetric study on radiation-induced hypothyroidism following intensity-modulated radiotherapy in patients with nasopharyngeal carcinoma |
| 28438291 | Zhai R | 2017 | 135 | Oral Oncol | Radiation-induced hypothyroidism after IMRT for nasopharyngeal carcinoma: Clinical and dosimetric predictors in a prospective cohort study |
| 35313921 | Zhai R | 2022 | 404 | Radiat Oncol | Predictors of radiation-induced hypothyroidism in nasopharyngeal carcinoma survivors after intensity-modulated radiotherapy |
| 32293496 | Zhou L | 2020 | 206 | Radiat Oncol | Thyroid V50 is a risk factor for hypothyroidism in patients with nasopharyngeal carcinoma treated with intensity-modulated radiation therapy: a retrospective study |
| 32461982 | Zhou L | 2020 | 206 | Biomed Res Int | Hematological Indexes Can Be Used to Predict the Incidence of Hypothyroidism in Nasopharyngeal Carcinoma Patients after Radiotherapy |
| 35154319 | Zhou L | 2022 | 170 | J Oncol | Association between Cervical Lymph Node Metastasis and the Incidence of Radiation-Induced Hypothyroidism in Nasopharyngeal Carcinoma |
| 34174518 | Zhu MY | 2021 | 244 | Oral Oncol | Radiation-induced hypothyroidism in patients with nasopharyngeal carcinoma treated with intensity-modulated radiation therapy with or without chemotherapy: Development of a nomogram based on the equivalent dose |
| 11917278 | Zoberi I | 2002 | Expected enrollement 100 | Semin Radiat Oncol | A prospective, nonrandomized study of the impact of amifostine on subsequent hypothyroidism in irradiated patients with head and neck cancers. |
| 6724961 | Zohar Y | 1984 | 144 | Head Neck Surg | Thyroid function following radiation and surgical therapy in head and neck malignancy. |
